# The cytological and molecular role of DOMAINS REARRANGED METHYLTRANSFERASE3 in RNA-dependent DNA methylation of *Arabidopsis thaliana*

**DOI:** 10.1186/1756-0500-7-721

**Published:** 2014-10-14

**Authors:** Pedro Costa-Nunes, Ji Young Kim, Evelyn Hong, Olga Pontes

**Affiliations:** Department of Biology, University of New Mexico, MSC03 2020, 1 University of New Mexico, Albuquerque, NM 87131 USA; Department of Biology, Washington University in St. Louis, 1 Brookings Drive, St. Louis, MO 63130 USA; Shanghai Center for Plant Stress Biology, Chinese Academy of Sciences, 3888 Chenhua Road, Shanghai, 201602 P. R. China

**Keywords:** Small RNAs, DNA methylation, DRM3, Nuclear localization, Interphase organization

## Abstract

**Background:**

Plants have evolved a unique epigenetic process to target DNA cytosine methylation: RNA-directed DNA methylation (RdDM). During RdDM, small RNAs (smRNAs) guide methylation of homologous DNA loci. In *Arabidopsis thaliana*, the *de novo* DNA methyltransferase that ultimately methylates cytosines guided by smRNAs in all sequence contexts is DOMAINS REARRANGED METHYLTRANSFERASE 2 (DRM2). Recent reports have shown that DRM2 requires the catalytic mutated paralog DRM3 to exert its function through a still largely unknown process. To shed light on how DRM3 affects RdDM, we have further characterized its role at the molecular and cytological levels.

**Findings:**

Although DRM3 is not required for RdDM loci transcriptional silencing, it specifically affects loci’s DNA methylation. Interestingly, DRM3 and DRM2 regulate the DNA methylation in a subset of loci differently.

Fluorescence In Situ Hybridization and immunolocalization analyses showed that DRM3 is not required for the large-scale nuclear organization of heterochromatin during interphase, with the notable exception of the 45S ribosomal RNA loci. DRM3 localizes exclusively to the nucleus and is enriched in a round-shaped domain located in the nucleolar periphery, in which it colocalizes with components of the RdDM pathway.

**Conclusions:**

Our analyses reinforce the previously proposed chaperone role of DRM3 in RdDM. Overall, our work further demonstrates that DRM3 most likely functions exclusively with DRM2 in RdDM and not with other *A. thaliana* DNA methyltransferases. However, DRM3’s regulation of DNA methylation is likely target- or chromatin context-dependent. DRM3 hypothetically acts in RdDM either upstream of DRM2, or in a parallel step.

**Electronic supplementary material:**

The online version of this article (doi:10.1186/1756-0500-7-721) contains supplementary material, which is available to authorized users.

## Findings

### Background

Eukaryotes modulate gene expression via histone post-translational modifications and/or DNA cytosine methylation. Repressive epigenetic marks, such as deacetylated histone tails and dimethylated lysine 9 of histone H3 (H3K9me2), are characteristic of silent chromatin, i.e. heterochromatin. Conversely, acetylated histones and H3 lysine 4 trimethylation (H3K4me3) are hallmarks of transcriptionally permissive chromatin, the euchromatic portion of the genome. In regards to DNA cytosine methylation, hypermethylation is usually associated with heterochromatin, while hypomethylation is associated with euchromatin.

In *Arabidopsis thaliana*, DNA methylation stably suppresses the activity of potentially deleterious transposons and other mobile elements throughout cellular division [1]. The *A. thaliana* genome encodes ten predicted cytosine methyltransferase genes, which can be clustered into three distinct groups according to their similarities with mammalian DNA methyltransferases [2]. METHYLTRANSFERASE1 (MET1) is the Dnmt1 ortholog responsible for maintenance methylation in CG motifs [3]. Symmetrical DNA methylation in the CHG context (in which H = A, T or C) is achieved by activity of CHROMOMETHYLASE3 (CMT3) [4], a member of the plant-specific CMT family. CMT2 is responsible for asymmetric methylation (CHH) at histone H1-enriched heterochromatic regions, binding to H3K9me2 [5]: a process facilitated by the chromatin remodeler DECREASED DNA METHYLATION 1 (DDM1) [6].

Finally, *A. thaliana* orthologs of the mammalian Dnmt3 family include DOMAINS REARRANGED METHYLTRANSFERASE1-3 (DRM1-3). DRM1 expression is restricted to the mature egg cell, where together with DRM2, it functions as a *de novo* methyltransferase [7]. 24-nucleotide (nt) short interfering RNAs (siRNAs) guide DRM2 activity in RNA-directed DNA methylation (RdDM), to methylate *de novo* asymmetric cytosines (CHH) [8, 9].

DRM3 is another member of the DRM family in *A. thaliana* that has been implicated in RdDM [10]. DRM3 is a catalytically mutated DRM2 paralog, as its Motif IV carries an S585N amino acid substitution. Intriguingly, although bearing a mutation in its catalytic domain, DRM3 is required to establish CHH DNA methylation at some RdDM target loci, such as *AtSNI*, *MEA-ISR* and *FWA*, and to accumulate 5S ribosomal RNA (rRNA) homologous siRNAs [10, 11]. Despite these effects, DRM3 cannot compensate for the loss of DRM2 and it is proposed that they function together at the step of catalysis to establish DNA methylation [10], in a mechanism not very well understood.

Given the importance of DNA methylation for regulating gene expression, which in turn affects organisms’ phenotype, the goal of this study was to deepen our understanding of how this epigenetic mark is established. Specifically, we sought to clarify the role of DRM3 in RdDM by determining whether DRM3 partners exclusively with DRM2 and how DRM3 relates to other fundamental components of the RdDM pathway. To that end, we characterized DRM3’s effects on several RdDM targets at both the transcriptional and DNA methylation levels. We also determined DRM3’s effect on nuclear organization during interphase, as well as its cellular localization.

## Results and discussion

### DRM3 is required for 24-nt siRNA accumulation but does not significantly contribute to transcriptional silencing at RdDM targets

As mentioned previously, DRM3 is proposed to act as a chaperone of DRM2 activity in RdDM [10], which is a complex process requiring several protein complexes. In short, siRNA biogenesis in *A. thaliana* results from the combined activity of the plant-specific RNA polymerase IV (Pol IV) and RNA-DEPENDENT RNA POLYMERASE 2 (RDR2) [12, 13]. Downstream of this process, the lead strand of the siRNA duplex is loaded to an Argonaute (AGO) protein, usually AGO4 [14, 15]. AGO4 can be crosslinked to noncoding transcripts of the plant-specific RNA polymerase V (Pol V), suggesting that co-transcriptional Pol V-dependent interactions account for AGO4-siRNA recruitment to target loci [16]. Finally, these AGO4-siRNA complexes, through interactions with other pathway members, direct DRM2 and possibly DRM3 activities [9, 10], resulting in the establishment of DNA methylation and repressive histone modifications to silence the corresponding genomic region [16, 17].

**Figure 1:**
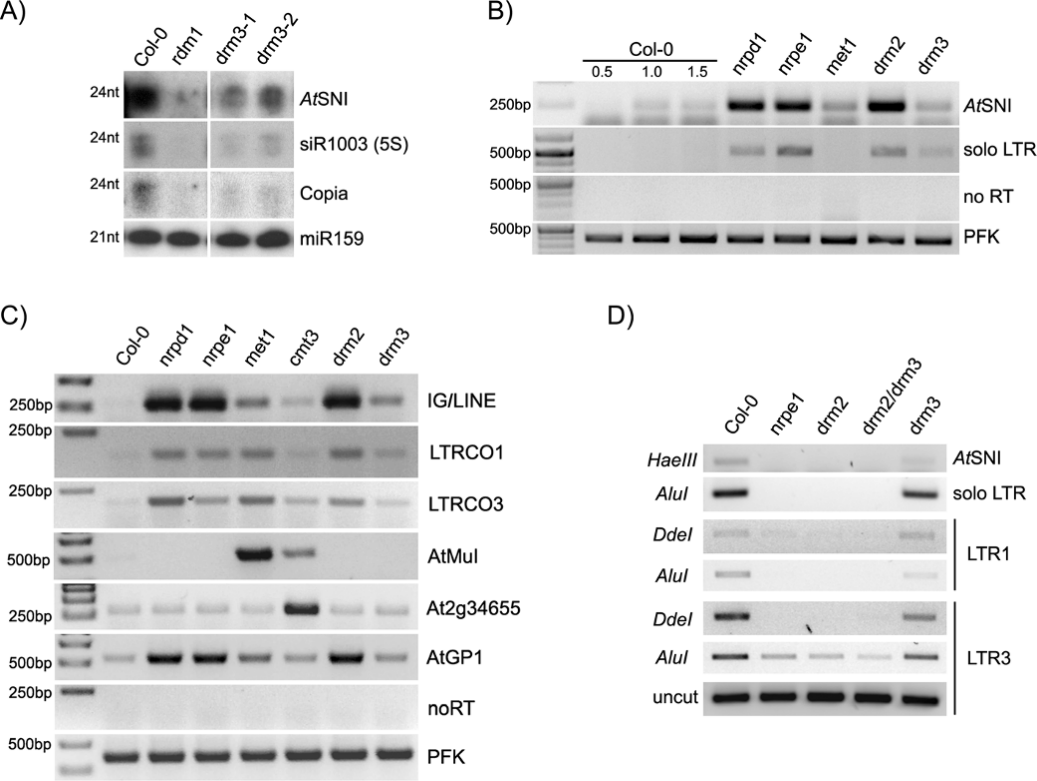
**Effect of** ***drm3*** **loss of function in siRNA accumulation, transcriptional activity and DNA methylation. (A)** DRM3 is required for the accumulation of repeat-derived 24-nt smRNAs. SmRNA Northern blot showing reduced 24 nt smRNA accumulation in a *drm3* mutant background relative to Col-0 (WT), as also observed for *rdm1* and *drm2*. **(B)(C)** RT-PCR analysis shows that the *drm3* mutation has only a minor effect on transcriptional reactivation of RdDM targets, when compared to other *bona fide* pathway members. Loci under the transcriptional control of MET1 or CMT3 do not require DRM3 activity (AtMuI, At2g34655). **(D)** Methylation Sensitive PCR (MSP) assay showing that CHH methylation is not severely compromised in loss of function *drm3* mutants relative to *drm2* and *nrpe1* mutants in RdDM-controlled loci.

To reveal whether other RdDM targets are also regulated by DRM3, we began by characterizing the effect of *drm3* on 24-nt siRNA production (Figure 1A) by RNA Northern blot. A significant reduction in its abundance relative to wild type (WT) was observed in 5S rRNA (siR1003), in agreement with [10]; similar reductions in *AtSNI* and Copia repeats were also observed.

In RdDM, loss of DNA methylation and siRNA production correlates with transcriptional reactivation at a given target locus. Semi-quantitative RT-PCR (Reverse Transcription Polymerase Chain reaction) of RdDM targets, such as *AtSNI*, *soloLTR* and *IG/LINE,* revealed that transcriptional reactivation occurs in *drm3* mutants at lower levels than in *nrpd1*, *nrpe1* (Pol IV and V’s largest subunits, respectively) or *drm2* genetic mutants (Figure 1B, C). Negligible effects on the transcriptional reactivation of *AtGP1*, *LTRCO1* and *LTRCO3* in *drm3* mutants were also observed (Figure 1C). Furthermore, DRM3 is not required for transcriptional regulation of *AtMuI*, *Athila*, *Ta3* and *CACTA* transposable elements (not shown) under MET1 control [18, 19], nor of the CMT3-regulated *At2g34655* loci (Figure 1C). These observations indicate that DRM3 is likely RdDM-specific, as according to our data, DRM3 is not required to silence MET1 and CMT3-dependent loci and because it functions together with DRM2 in RdDM dependent transcriptional silencing, as previously reported [10].

### DRM3 differentially affects DNA methylation at RdDM target loci

We next evaluated cytosine methylation at CHH sequence contexts by using a Methylation Sensitive PCR (MSP) assay. This assay allows the methylation status of cytosines to be evaluated at particular sequence motifs, by taking advantage of methylation-sensitive restriction enzymes. Methylated cytosines at the restriction enzyme recognition site will block endonuclease activity and allow for the PCR amplification, whereas DNA cleavage will occur at unmethylated sites and no PCR product will be observed. This MSP analysis revealed that for *AtSNI*, *solo LTR* and LTR1 and LTR3 regions of *LTRCO1* and *LTRCO3* transposons, which are single copy members of the *LTRCO* Copia-like retrotransposon family in *A. thaliana* [20], methylation was essentially unaffected in the *drm3* background (Figure 1D). In contrast, these transposable elements show extensive demethylation in *nrpe1*, *drm2* and the *drm2/drm3* double mutant at discrete cytosines (Figure 1D). Our MSP results correlate DNA demethylation with the transcriptional activity of RdDM targets (Figure 1B, C), with *drm3* displaying lower transcriptional reactivation levels than *nrpe1*, *nrpd1* and *drm2* mutants. DRM2 methylation at these loci was only slightly affected in a *drm3* background, implying that DRM2 does not strictly require DRM3 at all chromosomal locations.

**Figure 2:**
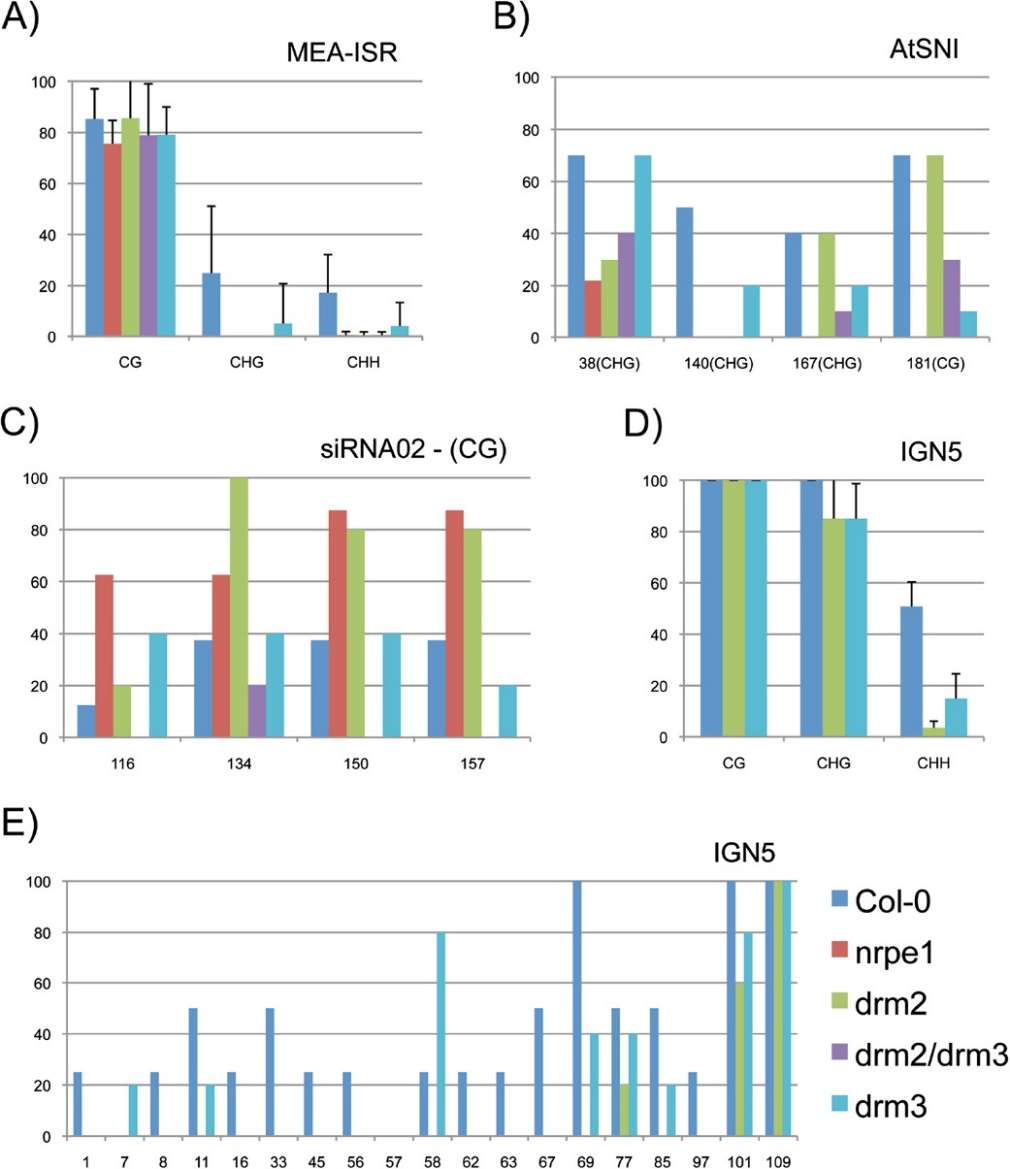
**Bisulfite sequencing analysis at distinct RdDM targets in** ***drm3*** **mutant. (A)** Frequency of cytosine methylation according to sequence context at *MEA-ISR* loci. A significant reduction of methylation at non-CG sites was observed in *drm3* relative to WT, but not to the levels observed for *drm2*. Frequency of symmetrical site methylation at **(B)** *AtSNI* and **(C)** siRNA02 (CG) are differentially affected by *drm2* and *drm3*. **(D)** Loss of CHH methylation at IGN5 is higher in a *drm2* background relative to *drm3*, especially at its 5’ end **(E)**. All cytosines are in a CHH context except at positions 101 (CHG) and 109 (CG) **(E)**. Where present, error bars refer to standard deviation. *y-axis*: frequency (%); *x-axis*: numbers correspond to cytosine position.

To obtain deeper insights into how DRM3 regulates DNA methylation, we performed bisulfite sequencing at RdDM-controlled loci, namely *simpleHAT2*, *MEA-ISR*, *AtSNI*, *siRNA02* and *IGN5*. No significant differences in cytosine methylation levels were found at the *simpleHAT2* locus between *drm2* and *drm3* (not shown). Our *MEA-ISR* bisulfite sequencing results (Figure 2A) are in agreement with Henderson et al. (2010): while *drm2* displayed 100% DNA methylation loss at CHH sites, the *drm3* mutant showed partial demethylation in 20% of the amplicons relative to WT (Additional file 1: Figure S1). Thus, the DRM3-dependent DNA methylation phenotype results from the differential regulation of a subset of target sequences at the *MEA-ISR* loci.

DNA methylation analysis of *AtSNI* loci by bisulfite sequencing showed a high degree of similarity between *drm2* and *drm3*, with an overall loss of CHH methylation and reduction at non-CHH sites (Additional file 1: Figure S2). However, a subset of non-CHH cytosines sites was decreased in the *drm3* and *nrpe1* mutant backgrounds, but not in *drm2* (Figure 2B). Also, marked differences between *drm2* and *drm3* were found at siRNA02 loci, where hypermethylation at CG sites was observed in *nrpe1* and *drm2,* but not in *drm3* nor in the *drm2/drm3* double mutant (Figure 2C). At IGN5, *drm2* and *drm3* mutations did not affect CG methylation and only slightly affected CHG sites compared to WT. In contrast, significantly fewer methylated cytosines were observed in a CHH context in both mutant lines (Figure 2D) relative to WT. Interestingly, while in the *drm2* background, CHH methylation was almost totally eliminated throughout the full length of the IGN5 sequence; in *drm3*, the 5’ end still displayed significant levels of DNA methylation compared to WT (Figure 2D, 2E). Taken together, these bisulfite analyses suggest that DRM3 affects DNA methylation differently than DRM2 at specific loci, and that this difference is likely influenced by chromosome/chromatin location and target loci.

### DRM3 associates with AGO4 at the nucleolar periphery

It has been previously shown that the RdDM pathway is localized in distinct nuclear and cytoplasmic subcompartments [21, 22]. For instance, Pol IV is strictly localized to the nucleoplasm, whereas RDR2 also accumulates in a distinct nuclear domain in the perinucleolar periphery [21, 23–25]. Proteins like RDM1, required for Pol V activity and targeting of DNA methylation, and AGO4 are also found in this nuclear domain. Importantly, mislocalization of the upstream RdDM components required for siRNA production and proper targeting of *de novo* DNA methylation affects the nuclear localization of downstream pathway members involved mainly in the targeting process [21]. In addition, active site mutants of Pol V’s largest subunit, NRPE1, abrogate DNA methylation at RdDM targets and disrupt the nuclear localization of the polymerase [26]. Together, this indicates that the cellular localization of RdDM pathway components is linked to their function. Therefore, determining the nuclear localization pattern of an RdDM pathway member and how its loss of function affects the nuclear organization of other proteins can yield important clues to determine at which step of the pathway it functions, namely, if it is required for siRNA biogenesis or involved in DNA methylation targeting [21].

**Figure 3:**
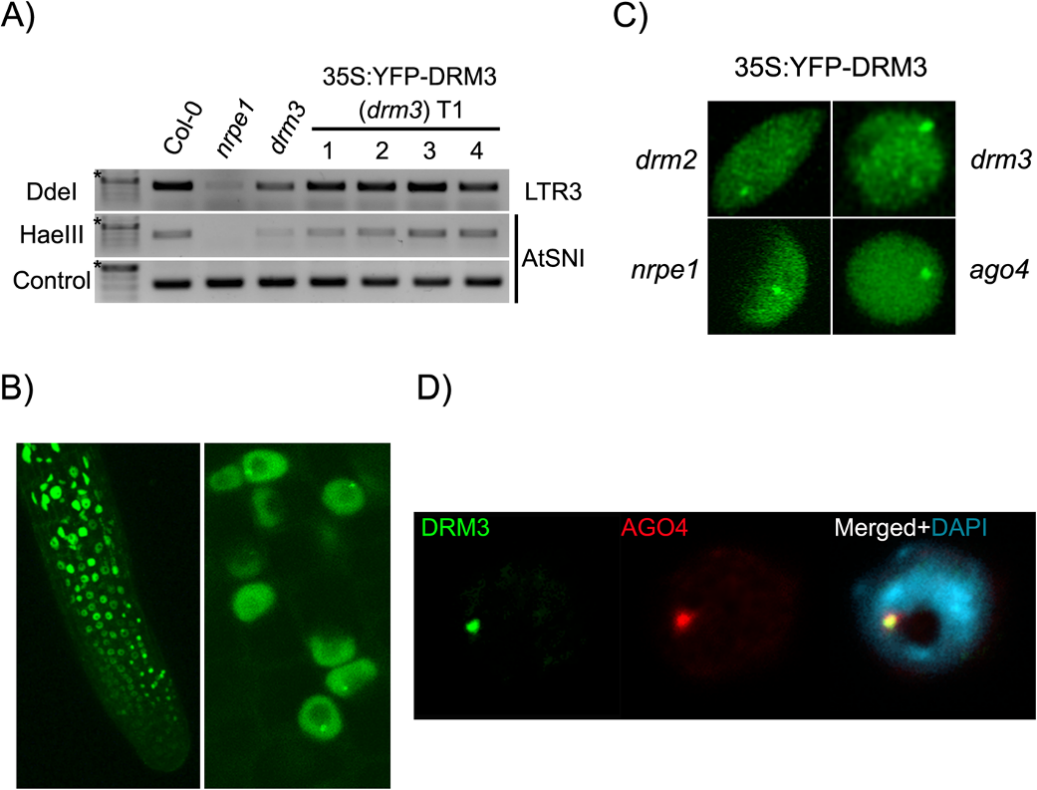
**DRM3 colocalizes with AGO4 in a nuclear domain in the nucleolar periphery. (A)** Methylation Sensitive PCR showing that the 35S:YFP-DRM3 transgene rescues the methylation phenotype of *drm3.* **(B)** Confocal microscope inspection showing transgene expression throughout the root (*left panel*) and the preferential accumulation of 35S:YFP-DRM3 in a round focus in the nucleolar periphery (*right panel*). **(C)** Confocal projections of root meristem nuclei indicate that interphase distribution of DRM3 does not depend on Pol V, AGO4 or DRM2 activity. **(D)** Immunolocalization of AGO4 (*red*) and DRM3 (*green*) show colocalization of both proteins in the smRNA perinucleolar body (>98%; n = 85). DNA counterstained with DAPI (*blue*). ★-500 bp.

To learn more about DRM3’s cellular localization and its relationship to other pathway members, we generated transgenic lines with an N-YFP DRM3 cDNA construct driven by a 35S promoter. The transgene was able to restore DNA methylation levels when introduced into a *drm3-1* background at both *AtSNI* loci [10] and in the LTR3 region, demonstrating that the transgene is functional (Figure 3A).

DRM3 localizes throughout the nucleoplasm and was found to accumulate preferentially in a perinucleolar focus (Figure 3B), in a pattern similar to the one observed for RdDM pathway members RDM1 and AGO4 [21, 23, 25]. The DRM3 interphase localization pattern was not disrupted when the transgene was introduced into *drm2*, *ago4* and *nrpe1* mutant backgrounds (Figure 3C), nor was the nuclear localization of AGO4 and DRM2 disrupted in a *drm3* background (Additional file 1: Figure S3). Interestingly, co-immunolocalization in interphase nuclei expressing YFP-DRM3 and AGO4, using an antibody specific to the native protein, showed that both proteins colocalized in a perinucleolar domain (Figure 3D), placing DRM3 in the hypothetical smRNA processing center previously described [21, 24].

Altogether, these observations suggest that DRM3 acts downstream of siRNA production in RdDM, as it is nonessential for the localization of the main components of RdDM. Nevertheless, due to its accumulation in the smRNA processing center, it is possible that DRM3 might act upstream of DRM2 in the pathway, in accordance with its proposed chaperone role for DRM2 [10]. The mammalian homologs of DRM2 and DRM3 (Dnmt3a and Dnmt3L, respectively) are known to interact, with the recruitment of Dnmt3a to target loci being dependent on the association of the non-catalytic Dnmt3L with chromatin [27, 28]. Given that DRM3 localizes with AGO4 in the same nuclear domain where other RdDM components are also enriched (e.g. RDM1) [25], one could speculate that DRM3 may interact with other proteins or be required for complex assembly.

### DRM3 is required for NOR nuclear organization during interphase

The spatial organization of the genome in the interphase nucleus depends on several factors, including DNA sequence, gene activity and external (a)biotic factors [29, 30]. Several studies have revealed that cytologically visible heterochromatin formation requires DNA methylation at CG sites and methylation of H3K9 maintained by MET1 and DDM1 [31, 32]. Within the nucleus, heterochromatin is usually visible as DAPI (4',6-diamidino-2-phenylindole)-bright domains – the chromocenters – enriched in repetitive sequences [33, 34]. In *ddm1* and *met1* nuclei, a reduction in the size of these chromocenters is observed as a result of the dispersion of pericentromeric sequences [33].

**Figure 4:**
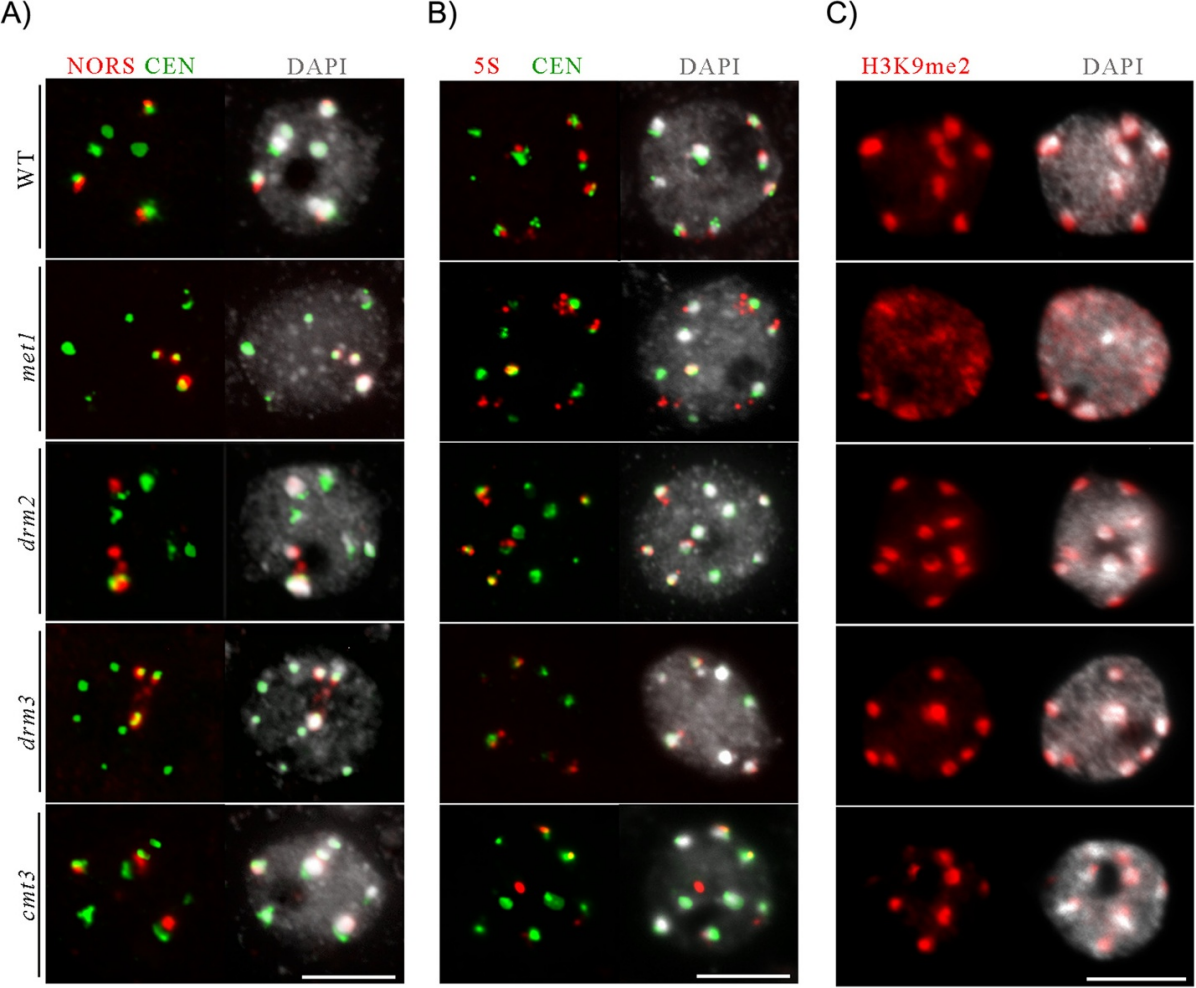
**DRM3 regulates NOR interphase organization. (A) DNA FISH showing decondensation of 45S rRNA loci (** ***red*** **) in** ***drm3*** **and** ***drm2*** **mutants.** Unlike *met1*, *drm3* is not required for chromocenter organization, as observed by WT interphase distribution of **(B)** CEN repeats (*green*), 5S rRNA loci (*red*) and **(C)** H3K9me2 in a *drm3* background. Representative images are shown for each genotype, corresponding to frequencies >91 %. (n > 64). DNA visualized with DAPI (*grey*). Size bar = 5 μm.

We evaluated whether DNA methylation regulated by DRM3 is correlated with spatial organization of heterochromatin within the nucleus. To that end, we used DAPI staining and Fluorescence In Situ Hybridization (FISH) to characterize the interphase organization of heterochromatic repeats in the *drm3* mutant background, when compared to *met1*, *cmt3* and *drm2* loss of function mutants (Figure 4). First, we measured the chromocenter fraction [35] in *met1*, *cmt3*, *drm2* and *drm3.* With the exception of *met1*, all analyzed DNA methyltransferase mutant lines displayed similar heterochromatin content as WT (not shown, see also Figure 3), indicating that DRM3-dependent cytosine methylation is not required for heterochromatin assembly during interphase. These observations are in agreement with previously published results showing that heterochromatin compaction is mainly dependent on MET1 activity [33, 36] and not on DRM2 [19].

Second, we used FISH with gene-specific probes to characterize the regulatory role of DRM3 in specific heterochromatic regions’ interphase organization (Figure 4). *A. thaliana* has four NOR loci located at the tips of chromosomes 2 and 4 [37, 38]. However, during interphase, NORs usually associate [35]; as a result, upon FISH analysis with a 45S rRNA DNA probe, three condensed foci, enclosed within chromocenters, were cytologically visible at the nucleolar periphery of the majority of the nuclei (65%; Figure 4A). In contrast, in both *drm3* and *drm2* mutant backgrounds, more nuclei with four NOR foci were observed (43% and 45%, respectively) relative to WT (30%) (see also Additional file 1: Table S1). In addition, the NOR’s FISH signals were smaller compared to WT nuclei, and partially located inside the nucleolus in both *drm2* and *drm3* null mutant backgrounds (red signals, Figure 4A, arrows). Upon FISH analysis with the 45S rRNA-specific probe, we observed no loss of association between NOR loci and the chromocenter in *met1* or *cmt3* mutants, nor foci decondensation.

In *A. thaliana* WT, 5S ribosomal DNA (rDNA) loci are located in the pericentromeric heterochromatin of chromosomes 3, 4 and 5 [39]. Both major and some minor 5S rRNA species from chromosomes 4 and 5 are expressed, whereas the genes on chromosome 3 are not [40]. It has been previously demonstrated that in leaf nuclei of mature WT plants, the transcribed fraction of 5S rDNA forms loops that emanate from chromocenters in a DDM1- and MET1-dependent manner [41, 42]. Our FISH analysis showed that 5S rRNA loci in *cmt3, drm3* and *drm2* displayed a WT interphase organization pattern, in contrast to the altered pattern observed in *met1* (Figure 4B; Additional file 1: Table S1), as previously reported [42]. These results demonstrate that, like DRM2, DRM3-directed methylation is not required for 5S rRNA loci interphase organization [43]. Likewise, FISH with probes specific to other heterochromatic repeat regions, like the 180-bp centromere repeats, did not detect an altered interphase organization pattern in *drm3* and *drm2* mutant backgrounds (red signals, Figure 4B; Additional file 1: Table S2).

Immunofluorescence analysis using antibodies specific to heterochromatic marks such as H3K9me2 (Figure 4C) and DNA methylation (not shown) revealed that *drm3*, *drm2* and *cmt3* nuclei display a WT nuclear organization, in which the H3K9me2 marks are enclosed in the chromocenters. In contrast, *met1* displayed a disrupted nuclear organization, with the H3K9me2 heterochromatic modification no longer confined to the chromocenters (Figure 4C; Additional file 1: Table S3).

In conclusion, DRM3 and DRM2 are both required for NOR loci nuclear organization but not for overall peri- and centromeric heterochromatin organization, in contrast to MET1. Our cytological observations strengthen the hypothesis that DRM3 acts together with DRM2 in RdDM and does not contribute to MET1 activity in the spatial assembly of pericentromeric heterochromatin.

## Materials & Methods

### Plant material and generation of transgenic lines

Loss of function mutants used in this study included *ago4-1* (At2g27040)*,* provided by Steve Jacobsen and in Landsberg background; *nrpd1-3* (At1g63020); *nrpe1-11* (At2g40030); *met1-1* (At5g49160)*,* provided by Eric Richards; *cmt3-11 t* (SALK_148381) (At1g69770); *rdm1-1* (At3g22680) provided by Jian-Kang Zhu; *drm3-1*, *drm3-2* (At3g17310) [10]; and *drm2-2* (At5g14620); all in Columbia ecotype (Col-0) background. Plants were grown in long-day conditions in a controlled environment (16 h light/8 h dark: 22°C) on soil or Murashige and Skoog medium with Gamborg vitamins (Sigma).

The full-length coding region of DRM3 was amplified by PCR and cloned into the pEarleyGate104 plant transformation vector using Gateway methodology (Invitrogen), as described previously [44]. The transgenic construct was subsequently transformed into the desired genetic background by the floral dip method [45].

### Fluorescence in situ hybridization (FISH), immunolocalization and microscopy

Leaf nuclei spreads were prepared according to [21]. Probe labeling for FISH was as follows: 45S rRNA probe was labeled with Biotin 16-dUTP following the Nick Translation Mix (Roche) protocol with the pAt.2 [46] plasmid as template; 5S rRNA (in pCT4.2) and 180 bp-CEN (in pARR20.1) probes were labeled by PCR with Biotin 16-dUTP and Digoxigenin-11-dUTP (Roche), respectively. FISH was performed as previously described [47] with a hybridization stringency of 81% (50% formamide: 2×SSC – at 37°C). Biotin-labeled probes were detected with streptavidin-Cy3 conjugate (1:300, Life Technologies). Digoxigenin-labeled probes were detected using mouse anti-digoxigenin antibody (1:250, Roche) followed by incubation with anti-mouse Alexa488 (Life Technologies). Immunolocalization of AGO4 was performed by making use of an antibody raised in rabbit against the native protein [16]. Immunofluorescence to detect H3K9me2 was performed with an anti-dimethyl-Histone H3 (Lys9) antibody (Millipore), as previously described [19]. DNA was counterstained with ProlongGold® antifade reagent with DAPI (Life Technologies). Preparations were inspected using a Zeiss AxioSkop2 mot plus upright microscope equipped with an AxioCam MRm camera. 35S:YFP-DRM3 nuclear localization was evaluated by live cell imaging using a Nikon A1 confocal microscope. Images were processed with AxioVision (Zeiss) and Adobe Photoshop software (Adobe Systems).

### DNA methylation analysis

DNA from 2-week-old leaves was extracted with NucleoSpin® Plant II (Macherey-Nagel), according to the manufacturer’s instructions. For MSP assays, 500 ng of DNA were digested with the selected restriction enzyme. Reactions without the selected restriction enzyme were also performed to serve as an input control. PCR was subsequently used to evaluate the cytosine methylation status at the targeted positions (Additional file 1: Table S4). Bisulfite conversion was performed with an EZ DNA Methylation-Lightning Kit™ (Zymo Research), with an input of 600 ng gDNA per sample. Modification efficiency was assessed by PCR amplification with a control primer pair [48]. Amplicons were generated from the target sequences, gel purified, cloned into pGEM®-T Easy (Promega) and transformed into bacteria. At least 10 individual clones were sequenced for each genotype per target sequence and sequencing results were analyzed with CyMATE [48]. See Additional file 1: Table S4 for a complete list of primers.

### Small RNA blots and transcriptional analysis

RNA was extracted from leaf tissue of 2-week-old plants following the enrichment procedure for smRNAs of the mirVana™ miRNA Isolation Kit (Ambion). The high molecular RNA fraction was digested with TURBO™ DNAse (Ambion). One μg of RNA was subsequently used for cDNA synthesis with Maxima Reverse Transcriptase (Thermo Scientific) and used as a template in RT-PCR analysis (see Additional file 1: Table S4).

For smRNA analysis, 10 μg of the low molecular weight fraction were separated on a 20% polyacrylamide denaturing gel (7 M urea; 50 mM TBE) and transferred on a semi-dry blotter, 2.0 mA/cm^2^ for 2 h, to a MAGNACHARGE nylon membrane (0.22 μm) (GE Osmonics). RNA probes were radioactively labeled with a mirVana™ miRNA Probe Construction Kit (Ambion) according to manufacturer’s instructions (Additional file 1: Table S4). RNA hybridization was performed as previously described [49].

## Electronic supplementary material

Additional file 1: Figure S1: Cymate (cymate.org) output file showing methylation status of cytosines in *MEA-ISR* loci for the analyzed mutant lines. Red arrows highlight amplicons displaying partial loss of CNN methylation relative to WT. **Figure S2.** Mapping and comparison of cytosine methylation frequency of *AtSNI* repeats between WT Col-0, *nrpe1*, *drm2*, *drm3* and *drm2/drm3* double mutant lines by bisulfite sequencing. **Figure S3.** (A) AGO4 interphase localization is not dependent of DRM3. No alteration of the immunolocalization patterns of AGO4 in WT and *drm3* mutant line was observed. (B) Confocal projection showing DRM3-YFP localizing to the small RNA processing center. DRM3-YFP localization is not disrupted in a *drm2* mutant background (compare left panels). Likewise, no alteration to the nucleoplasmic DRM2-GFP interphase localization was observed in a *drm3* background relative to WT (compare right panels). **Table S1.** – Frequency (%) of nuclei displaying different numbers of NORs in different DNA methylation mutant backgrounds. **Table S2.** – Frequency (%) of nuclei displaying different numbers of centromere foci during interphase in DNA methyltranferase mutants. **Table S3.** - Frequency (%) of nuclei displaying co-localization of H3K9met immunostaining signals with chromocenters in DNA methyltransferease mutants. **Table S4.** Primer List. Supplementary references. (PDF 3 MB)
