# Supplementary material for: The cytological and molecular role of DOMAINS REARRANGED METHYLTRANSFERASE3 in RNA-dependent DNA methylation of Arabidopsis thaliana
Source: BMC Res Notes. 2014 Oct 14;7:721. doi: 10.1186/1756-0500-7-721 (PMC4209038; doi:10.1186/1756-0500-7-721)
Supplement: Supplementary file 1 — Additional file 1: Figure S1: Cymate (cymate.org) output file showing methylation status of cytosines in MEA-ISR loci for the analyzed mutant lines. Red arrows highlight amplicons displaying partial loss of CNN methylation relative to WT. Figure S2. Mapping and comparison of cytosine methylation frequency of AtSNI repeats between WT Col-0, nrpe1, drm2, drm3 and drm2/drm3 double mutant lines by bisulfite sequencing. Figure S3. (A) AGO4 interphase localization is not dependent of DRM3. No alteration of the immunolocalization patterns of AGO4 in WT and drm3 mutant line was observed. (B) Confocal projection showing DRM3-YFP localizing to the small RNA processing center. DRM3-YFP localization is not disrupted in a drm2 mutant background (compare left panels). Likewise, no alteration to the nucleoplasmic DRM2-GFP interphase localization was observed in a drm3 background relative to WT (compare right panels). Table S1. – Frequency (%) of nuclei displaying different numbers of NORs in different DNA methylation mutant backgrounds. Table S2. – Frequency (%) of nuclei displaying different numbers of centromere foci during interphase in DNA methyltranferase mutants. Table S3. - Frequency (%) of nuclei displaying co-localization of H3K9met immunostaining signals with chromocenters in DNA methyltransferease mutants. Table S4. Primer List. Supplementary references. (PDF 3 MB) [file 13104_2014_3250_MOESM1_ESM.pdf]

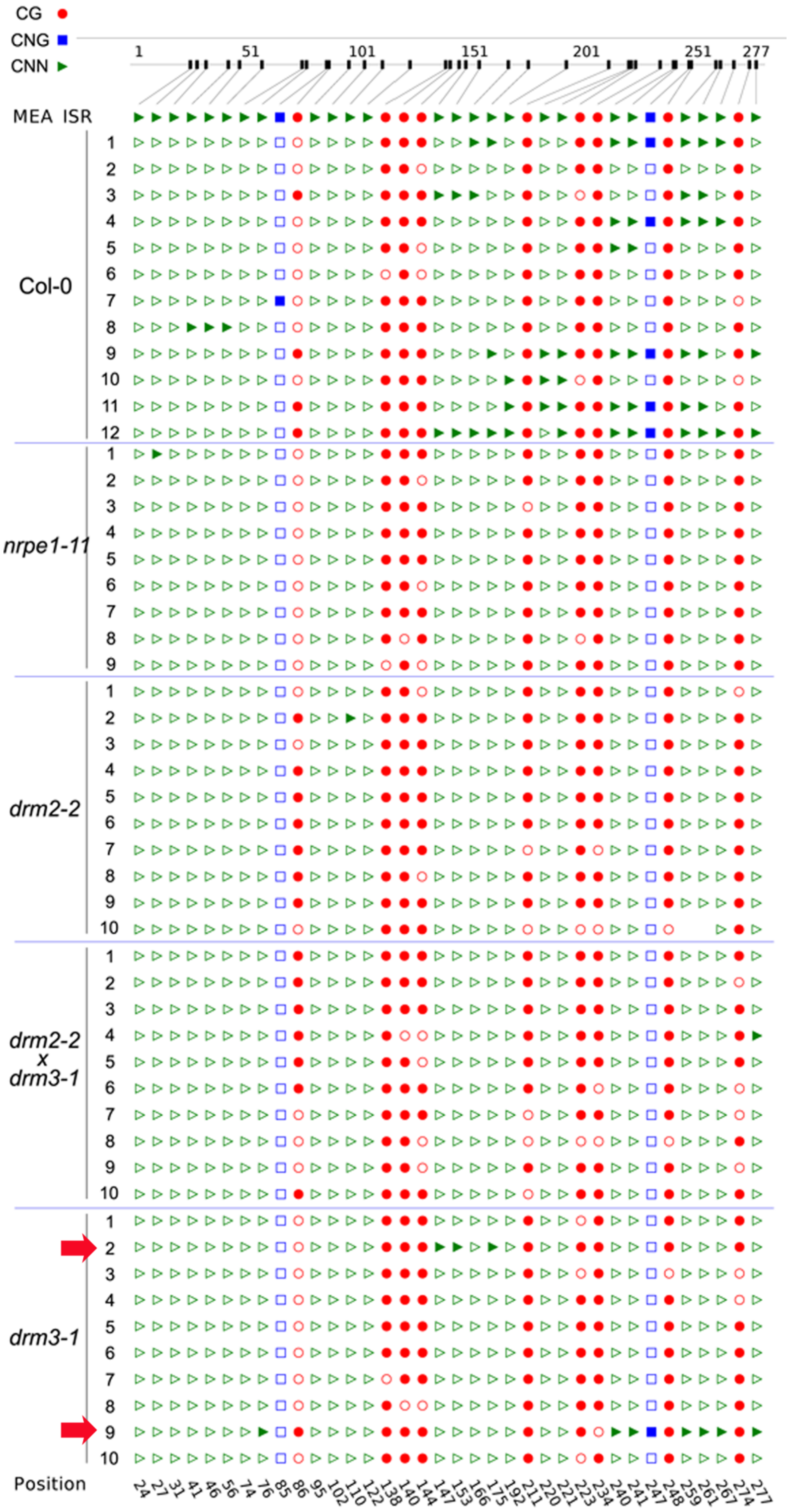

Supplemental figure 1: Cymate (cymate.org) output file showing methylation status of cytosines in *MEA-ISR* loci for the analyzed mutant lines. Red arrows highlight amplicons displaying partial loss of CNN methylation relative to WT.

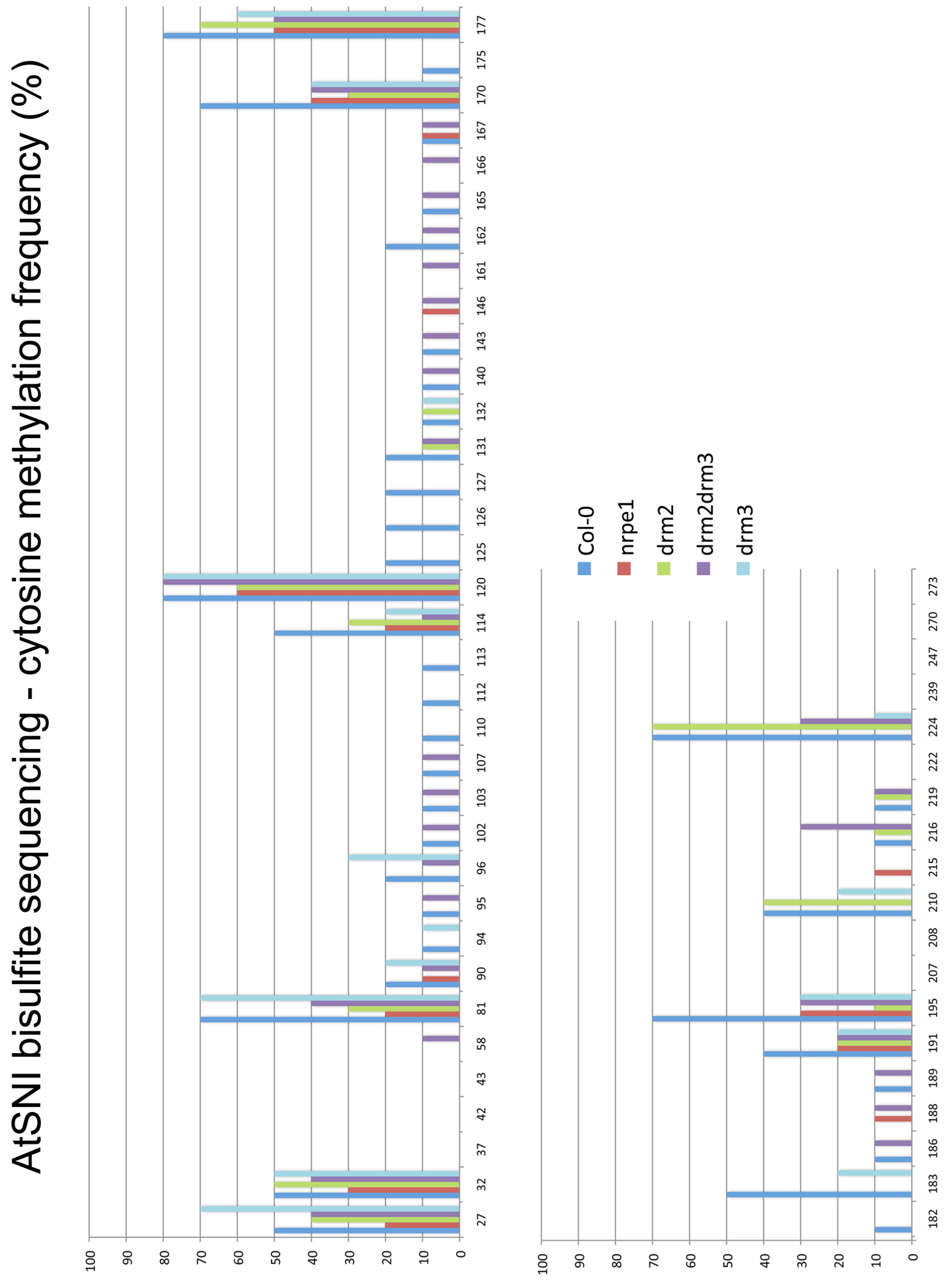

Supplemental figure 2: Mapping and comparison of cytosine methylation frequency of *AtSNI* repeats between WT Col-0, *nrpe1*, *drm2*, *drm3* and *drm2/drm3* double mutant lines by bisulfite sequencing.

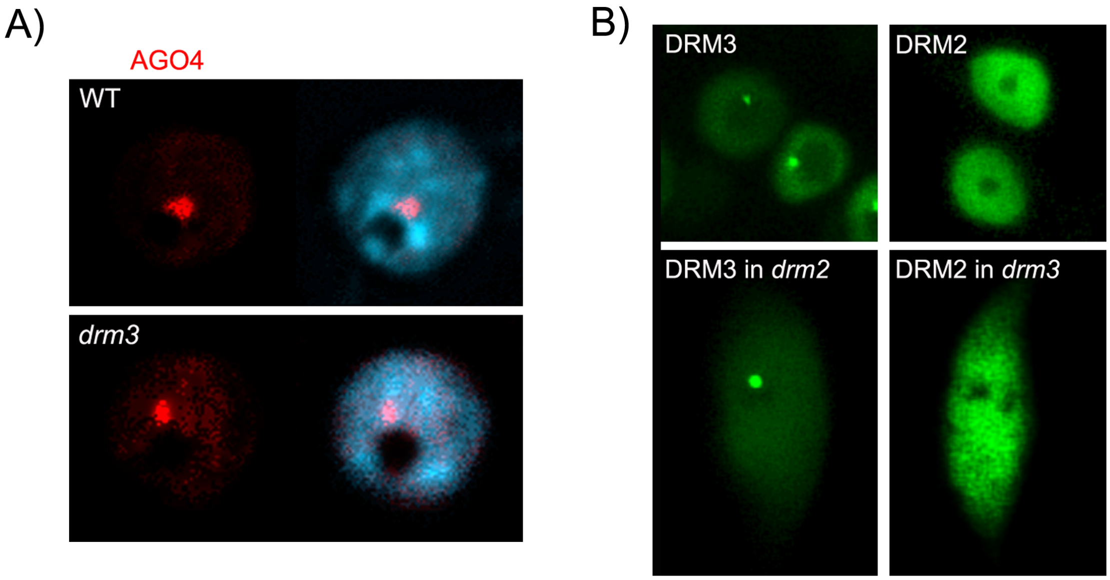

Supplemental figure 3: (A) AGO4 interphase localization is not dependent of DRM3. No alteration of the immunolocalization patterns of AGO4 in WT and *drm3* mutant line was observed. (B) Confocal projection showing DRM3-YFP localizing to the small RNA processing center. DRM3-YFP localization is not disrupted in a *drm2* mutant background (compare left panels). Likewise, no alteration to the nucleoplasmic DRM2-GFP interphase localization was observed in a *drm3* background relative to WT (compare right panels).

Supplemental table 1 – Frequency (%) of nuclei displaying different numbers of NORs in different DNA methylation mutant backgrounds

	**Number of NOR *foci***				
	**4**	**3**	**2**	**P**	**n**
**Col-0**	30	65	5		96
drm3	43	47	10	0.0004*	125
drm2	45	43	12	0.0001*	135
met1	32	63	5	0.9072	87
cmt3	26	66	8	0.309	72

Note that *drm2* and *drm3* are not statistically different (p=0.6722) from one another - ChiSquare 2 degrees of freedom. (*) Statistically significant.

Supplemental table 2 – Frequency (%) of nuclei displaying different numbers of centromere foci during interphase in DNA methyltranferase mutants.

	**Number of Centromere *foci***			
	**7-10**	**>10**	**P**	**n**
**Col-0**	82	8		68
drm3	83	7	1.0000	76
drm2	79	11	0.6286	85
met1	43	67	>0.0001*	125
cmt3	85	5	0.5664	140

Two tailed Fisher's exact test. (*) Statistically significant.

Supplemental table 3 - Frequency (%) of nuclei displaying co-localization of H3K9met immunostaining signals with chromocenters in DNA methyltransferease mutants.

	**colocalized**	**not-colocalized**	**P**	**n**
**Col-0**	71	29		180
drm3	67	33	0.6467	131
drm2	73	27	0.857	125
met1	56	44	0.0394*	205
cmt3	69	31	0.8775	91

Two tailed Fisher's exact test. (*) Statistically significant.

Supplemental table 4

DNA oligos

**smRNA probes**		
AtSNI	CCTCTATCTGAGAGATTTACCACTGGGCCAACACGTTGGCcttgtctc	Pontes el. 2006
siR1003	AGACCGTGAGGCCAAACTTGGCATcttgtctc	Pontes el. 2006
Copia	TTATTGGAACCCGGTTAGGActtgtctc	Pontes el. 2006
miR159	TTTGGATTGAAGGGAGCTCTACcttgtctc	Pontes el. 2006
**RT-PCR primers**		
IG/LINE Fwd	AACTAACGTCATTACATACACATCTTG	Huettel et al. 2006
IG/LINE Rev	AATTAGGATCTTGTTTGCCAGCTA	Huettel et al. 2006
LTRCO1 fwd	CGAATGGTAAGCTATGCCT	Huettel et al. 2006
LTRCO1 rev	TTAATATCTGATGCCCATGACT	Huettel et al. 2006
LTRCO3 fwd	CGAATGGTAGGCTATGCTA	Huettel et al. 2006
LTRCO3 rev	AATATCTGATGCCCATGACA	Huettel et al. 2006
soloLTR fwd	AATGCATTACAAAAACCTTCTGA	Huettel et al. 2006
soloLTR rev	GGATTCACGATTAGAGAACGTAGA	Huettel et al. 2006
At2g34655_RT1	TAT GTG CGT TGT GGT GTA GG	
At2g34655_RT2	CGA ATC AAA GAA TAT GAT TGT TTA GCG	
AtSNI-RT fwd	ACCAACGTGCTGTTGGCCCAGTGGTAAATC	Herr et al. 2005
AtSNI-RT rev	AAAATAAGTGGTGGTTGTACAAGC	Herr et al. 2005
AtMuI fwd	GTGGATATACCAAAAACACAA	He et al. 2009
AtMuI rev	CTTAGCCTTCTTTTCAATCTCA	He et al. 2009
AtGP1 fwd	ACAGTGCCACAGTTGAGCAG	He et al. 2009
AtGP1 rev	CAGAAAAAATACTCGGTGCCAAT	He et al. 2009
PFK fwd	CGC CGG AAT TTC GAT CAT CCT	
PFK rev	CGC CAC GAA AAC CAA ACA GAC	
**Bisulfite Sequencing**		
SIMPLEHAT2-Bis R	TAGGGGTGTTAAAATGGGTTAAAATTT	Zheng et al. 2007
SIMPLEHAT2-Bis F	ATAAAAAAATTACGAATTTACTTTTTCTC	Zheng et al. 2007
siRNA02_Bis F	ATY ATA TTT AGA TAG ATA GGA GAA AG	Zheng et al. 2007
siRNA02_Bis R	AAR AAT ATR CTA ATT TTT CTA TCA TA	Zheng et al. 2007
IGN5 Bis F	GTTYYYGAGAAGAGTAGAAYAAATGYTAAAATGTATYATGYGGTT	Zheng et al. 2007
IGN5 Bis R	RRACTAARTCTTRTCRAACAARRACCCAACCATRTCCRCTTAAAAA	Zheng et al. 2007
BScontrol1Fwd	CGT CTG GTG ATT CAC CCA CTT CTG TTC TCA ACG	Hetzl et al. 2007
BSconverted2Fwd	TGT TTG GTG ATT TAT TTA TTT TTG TTT TTA ATG	Hetzl et al. 2007
Bscontrol Rev	CTC TCA CTT TCT ATC CCA TTC TA	Hetzl et al. 2007
AtSNI-Bis fwd	GTTGTATAAGTTTAGTTTTAATTTTAYGGATYAGTATTAATTT	Henderson et al. 2006
AtSNI-Bis rev	CAATATACRATCCAAAAAACARTTATTAAAATAATATCTTAA	Henderson et al. 2006
MEA-ISR-Bi-F	AAAGTGGTTGTAGTTTATGAAAGGTTTTAT	Henderson et al. 2006
MEA-ISR-Bi-R	CTTAAAAAAATTTTCAACTCATTTTTTTTAAAAAA	Henderson et al. 2006
**Methylation Sensitive PCR**		
AtSN1-Methyl 5F	ACTTAATTAGCACTCAAATTAAACAAAATAAGT	Herr et al. 2005
AtSN1-Methyl 3R	TTTAAACATAAGAAGAAGTTCCTTTTTCATCTAC	Herr et al. 2005
LTR1 fwd	ATTAACCACACGCTGCATCTAAT	Huettel et al. 2006
LTR1 rev	GAGAGACGATCGTGCTGATAAC	Huettel et al. 2006
LTR3 fwd	GCAGTTAATTGTAAAATGACTTATGAA	Huettel et al. 2006
LTR3 rev	AGACGATCGTGCTGATAACG	Huettel et al. 2006
soloLTR Met fwd	AATGCATTACAAAAACCTTCTGA	Huettel et al. 2006
solo LTR Met rev	GGATTCACGATTAGAGAACGTAGA	Huettel et al. 2006

Supplementary References

Zheng X, Zhu J, Kapoor A, Zhu JK: **Role of Arabidopsis AGO6 in siRNA accumulation, DNA methylation and transcriptional gene silencing.** *EMBO J* 2007, **26**: 1691-1701.

Henderson I, Zhang X, Lu C, Johnson L, Meyers BC, Green PJ, Jacobsen SE: **Dissecting Arabidopsis thaliana DICER function in small RNA processing, gene silencing and DNA methylation patterning.** *Nat Genet* 2006, **38**: 721-725.

Herr AJ, Jensen MB, Dalmay T, Baulcombe DC: **RNA polymerase IV directs silencing of endogenous DNA.** *Science* 2005, **308**: 118-120.

He XJ, Hsu YF, Zhu S, Liu HL, Pontes O, Zhu J, Cui X, Wang CS, Zhu JK: **A conserved transcriptional regulator is required for RNA-directed DNA methylation and plant development.** *Genes Dev* 2009, **23**: 2717-2722.
